# Management of Graves Thyroidal and Extrathyroidal Disease: An Update

**DOI:** 10.1210/clinem/dgaa646

**Published:** 2020-09-14

**Authors:** George J Kahaly

**Affiliations:** Department of Medicine I, Johannes Gutenberg University (JGU) Medical Center, Mainz, Germany

**Keywords:** Graves disease, management, diagnosis, treatment, TSH receptor antibodies, antithyroid drugs, radioactive iodine, thyroidectomy

## Abstract

**Context:**

Invited update on the management of systemic autoimmune Graves disease (GD) and associated Graves orbitopathy (GO).

**Evidence acquisition:**

Guidelines, pertinent original articles, systemic reviews, and meta-analyses.

**Evidence synthesis:**

Thyrotropin receptor antibodies (TSH-R-Abs), foremost the stimulatory TSH-R-Abs, are a specific biomarker for GD. Their measurement assists in the differential diagnosis of hyperthyroidism and offers accurate and rapid diagnosis of GD. Thyroid ultrasound is a sensitive imaging tool for GD. Worldwide, thionamides are the favored treatment (12-18 months) of newly diagnosed GD, with methimazole (MMI) as the preferred drug. Patients with persistently high TSH-R-Abs and/or persistent hyperthyroidism at 18 months, or with a relapse after completing a course of MMI, can opt for a definitive therapy with radioactive iodine (RAI) or total thyroidectomy (TX). Continued long-term, low-dose MMI administration is a valuable and safe alternative. Patient choice, both at initial presentation of GD and at recurrence, should be emphasized. Propylthiouracil is preferred to MMI during the first trimester of pregnancy. TX is best performed by a high-volume thyroid surgeon. RAI should be avoided in GD patients with active GO, especially in smokers. Recently, a promising therapy with an anti-insulin-like growth factor-1 monoclonal antibody for patients with active/severe GO was approved by the Food and Drug Administration. COVID-19 infection is a risk factor for poorly controlled hyperthyroidism, which contributes to the infection–related mortality risk. If GO is not severe, systemic steroid treatment should be postponed during COVID-19 while local treatment and preventive measures are offered.

**Conclusions:**

A clear trend towards serological diagnosis and medical treatment of GD has emerged.

This mini-review offers an update on the diagnosis and management of patients with thyroidal and extrathyroidal Graves disease (GD). The purpose is to avoid repeating known and general knowledge. Hence, this update emphasizes the recent developments, for example, the serological accurate and rapid diagnosis of GD as well as its worldwide acknowledged favored medical treatment. It reports on the insulin-like growth factor-1 receptor (IGF-1R) as novel autoantigen in GD and associated Graves orbitopathy (GO). Further, this short review introduces novel, more disease-specific treatments for both GD and GO with special focus on the recently FDA-approved IGF-1R inhibiting monoclonal antibody teprotumumab. Finally, the management of GD and GO in the time of the COVID-19 pandemic is discussed.

The prevalence of GD approximates 1% to 1.5% in the population as a whole: the incidence is 20 to 30 new cases/100 000/year (1-4). An increased incidence is observed among African Americans (5). Both genetic and environment factors, for example, familial clustering, negative life experiences, high iodine intake, and smoking, predispose to GD (6-13). GD is a systemic autoimmune disease directly caused by circulating autoantibodies (Abs) that bind to the thyrotropin receptor (TSH-R), subsequently inducing the production and release of thyroid hormone, proliferation of thyrocytes, and enlargement of the thyroid gland (14-17). The stimulatory TSH-R-Ab (TSAb) is the causative agent in GD in patients suspected of having hyperthyroidism, with associated signs and symptoms (17). TSAb increases intracellular cyclic adenosine 5′-monophosphate directly when in contact with the TSH-R and can be detected in a bioassay using chemiluminescence (18). Further, TSAbs directly and specifically induce oxidative stress in GD (15).

## Methodology

Pertinent and current guidelines, original articles, clinical trials, systemic reviews, and meta-analyses were searched by using the following terms: “diagnosis of Graves’ disease,” “imaging of Graves’ disease,” “management of Graves’ disease,” “treatment of autoimmune hyperthyroidism,” “autoimmune thyrotoxicosis,” antithyroid drugs, thionamides, radioactive iodine, thyroidectomy, “Graves’ orbitopathy and/or ophthalmopathy,” “thyroid eye disease,” and “extrathyroidal Graves’ manifestations.”

## Diagnosis

### Serology

The measurement of serum baseline TSH is the acknowledged screening parameter in the initial evaluation of suspected thyroid dysfunction (19-21). Additional assessment of the free thyroid hormones differentiates between overt and subclinical hyperthyroidism (21).

**Figure 1.**
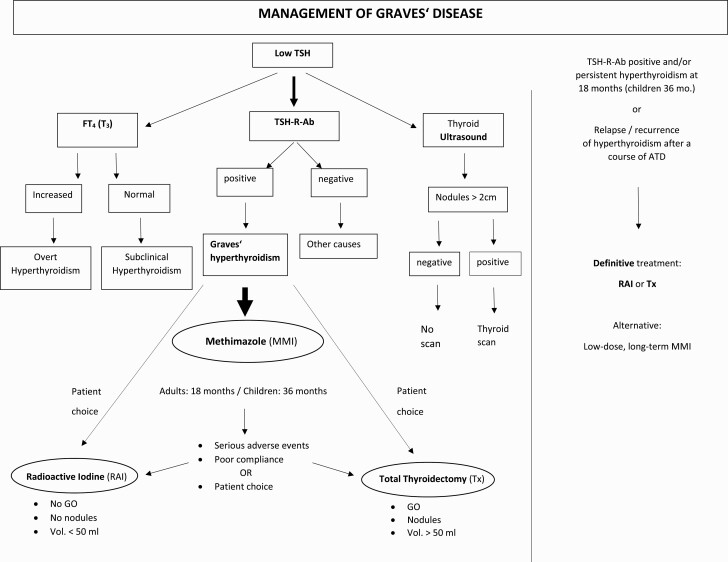
Algorithm for the management of Graves hyperthyroidism. TSH-R-Ab, thyrotropin receptor antibodies; FT4, free thyroxine; T3, triiodothyronine; scan, thyroid scintigraphy; MMI, Methimazole; RAI, radioactive iodine; TX, total thyroidectomy; GO, Graves orbitopathy; mo., months.

TSH-R-Ab measurement offers accurate and fast diagnosis of GD (Fig. 1) (3, 22, 23). The conventional TSH-R-Ab binding immunoassays are sensitive and specific (98 and 99%, respectively); however, these assays do not differentiate between Ab functionality (24, 25). In comparison, the cell-based bioassays measure TSH-R-Ab concentrations at very low Ab concentration and at very high serum dilution (26-33), and exclusively report on TSH-R-Ab functionality (34-36). Indeed, the bioassays show the functionality of the TSH-R-Abs that interact with the human TSH-R, and can specifically detect TSAbs, differentiating between the TSH-R-Ab types (blocking/stimulating). Functional TSH-R-Abs are also predictive for extrathyroidal manifestations (37-43) and for neonatal thyroid dysfunction (44-46). Further to their clinical utility and diagnostic relevance, measurement of stimulatory TSH-R-Abs markedly reduces both costs and time to diagnosis of GD by 50% (47).

### Imaging

Thyroid ultrasound comprises conventional gray-scale analysis (using a high-frequency linear probe) and color flow Doppler examination (characterizing vascular patterns and quantifying thyroid vascularity). Ultrasound supports the diagnosis of GD as a noninvasive, rapid, and accurate imaging procedure. If a goiter with large nodules is present and/or if radioactive iodine (RAI) therapy is indicated, radionuclide scintigraphy with radioiodine uptake is suggested (48-52). In the vast majority of cases, a timely and positive TSH-R-Ab serology with a typical ultrasound finding offers a reliable, rapid, and more than sufficient definitive diagnosis of GD.

## Treatment

Three equally potent approaches are offered to treat Graves hyperthyroidism: medical therapy by inhibiting production of thyroid hormones, complete surgical removal of the thyroid gland (total thyroidectomy), or RAI-induced shrinkage of the thyroid tissue (Fig. 1 and Table 1). The importance of patient choice, in a shared decision-making process, both at the initial episode of hyperthyroidism and at recurrence, should be emphasized.

**Table 1.** Advantages and disadvantages of the current established treatments of Graves disease

Treatment	Mechanism of action	Advantages	Disadvantages	Major adverse events
Antithyroid thionamide drugs	Inhibit thyroid hormone synthesis PTU blocks T4:T3 conversion	No radiation exposure No adverse effect on GO No risk of surgery/anesthesia No hospitalization required Conservative treatment Use during pregnancy/breastfeeding Low risk of subsequent hypothyroidism	High relapse rate Frequent monitoring Adverse events (rarely major) Compliance warranted	Agranulocytosis Hepatotoxicity Vasculitis Pancreatitis (?)
RAI	RAI-induced thyrocyte destruction	Definitive treatment High efficacy No risk of surgery/anesthesia Moderate costs	Definite risk factor for GO Slow control of hyperthyroidism Lifelong hypothyroidism Radiation exposure Pregnancy and breast feeding constitute absolute contraindications to RAI therapy Conception should be postponed until at least 6 (better 12) months after RAI in both males and females. Potential negative impact on fertility	Frequent de novo or exacerbation of pre-existing GO Sialadenitis (short-term) Radiation thyroiditis Enhanced cancer mortality (?)
Total thyroidectomy	Removal of thyroid gland	Definitive treatment Highest efficacy (experienced surgeon) Rapid control of hyperthyroidism Does not usually worsen GO No radiation exposure	High-volume surgeon recommended Risks related to anesthesia and/or surgery Permanent hypothyroidism Hospitalization High costs Scar	Bleeding Laryngeal nerve injury Hypoparathyroidism Hypocalcemia Anesthesia complications

Abbreviations: RAI, radioactive iodine; PTU, propylthiouracil; GO, Graves orbitopathy.

## Antithyroid Drugs

The thionamide antithyroid drugs (ATDs) have been the most common treatment of GD in Asia, Europe, and Latin America, in contrast to the USA where RAI has been more prevalent (22, 53). More recently, however, ATDs have been used in nearly 60% of GD patients in the USA versus 35% only for RAI (54). This is mainly because RAI is associated with both sustained increases in TSH-R-Ab titers after RAI and a definite risk for a de novo GO and/or exacerbation of the clinical activity and severity of the orbital disease (55-62).

The thiourea derivatives (ATDs) inhibit iodine organification, a process catalyzed by thyroid peroxidase (63, 64). ATDs negatively impact the activity and numbers of intrathyroidal T cells (65, 66), the aberrant thyrocyte expression of major histocompatibility complex class II (67), as well as the generation of reactive oxygen species, lipid peroxidation, and subsequent oxidative damage (15, 68-70).

Three compounds are available with methimazole (MMI) as the classical and most widely distributed ATD. In comparison, carbimazole (CBM) is an inactive drug and much less available worldwide. CBM is rapidly metabolized in the blood to MMI with an average 2-fold weaker potency than MMI (71). Propylthiouracil (PTU) is the least potent compound (10-fold weaker than MMI). However PTU additionally inhibits the thyroxine (T4) deiodination to 3,5,3′-triiodothyronine (T3) in peripheral tissues (72, 73). MMI has the longest half-life, an acceptable and low side-effect profile and is, therefore, regarded as the standard ATD with the highest efficacy (74, 75). Absorption of MMI following oral administration approximates 100% leading to peak serum levels within 1 to 2 hours. The intrathyroidal concentration of MMI is markedly higher than in the plasma with stable intrathyroidal levels for approximately 22 hours postresorption (76). Initial daily dosing of MMI depends on the severity of hyperthyroidism and is based on the elevation in free thyroid hormone levels; using 20 and 30 mg if fT4 is 2- and 3-fold increased, respectively (2, 14). The inhibition of the T4 deiodination and the lower placental transfer and less severe PTU-caused embryopathy explain why PTU is the first-line therapy of both thyroid storm in GD as well as in the first 10 to 12 weeks of pregnancy (77-79).

Overall, patients with newly diagnosed GD are offered 3 equally effective treatments; however, ATDs are by far the patient-preferred approach worldwide (2, 3). An ATD, preferably MMI, is administered for 12 to 18 months according to the American and European guidelines (1-3, 14, 80). Discontinuation of ATD therapy is indicated after reaching both biochemical euthyroidism (thyroid-related hormones within the normal range) and serological conversion (negative TSH-R-Ab testing). Indeed, the measurement of TSH-R-Ab levels in general and stimulatory TSH-R-Abs in particular is clinically useful at 18 months and predictive of recurrence (persistent stimulatory TSH-R-Ab) or remission (negative TSH-R-Ab). The European guidelines recommend the continuation of a low-dose MMI administration for a further 12 months with repeated TSH-R-Ab testing after 1 year (2, 14). Alternatively, definitive treatment with either RAI or TX may be considered (1, 2, 14) (Fig. 1). The evidence-based titration regimen with MMI monotherapy is favored since the addition of levothyroxine to MMI (block and replace regimen) neither enhances the remission rate nor decreases the recurrence of hyperthyroidism (81-84). Only about 50% of patients receiving ATD for 12 to 18 months will achieve a permanent remission, namely biochemical euthyroidism and negative TSH-R-Abs (85). The magnitude and presence of serum TSH-R-Ab levels, but also goiter size, the severity of hyperthyroidism, nicotine, young age, and the postpartum period negatively impact the recurrence rate (75, 86-89). Due to the low predictive value of each of the above risk factors, additional cumulative scores taking into account 4 baseline characteristics (TSH-R-Abs, fT4, goiter size, and age) have been proposed and were shown to provide suboptimal differentiation (90, 91).

### Adverse events

The most common adverse events (AEs) ascribed to ATD include minor cutaneous allergic reactions, including rash (3-6%), pruritus (2-3%), and urticaria (1-2%), as well as dyspepsia (3-4%), nausea/gastric distress (2.4%), and arthralgia (1.6%). Cutaneous reactions to ATD occur early during therapy (92). Major AEs include agranulocytosis, hepatotoxicity, vasculitis, and discordant reports on pancreatitis (74, 93-95). Agranulocytosis occurs in 0.2% to 0.5% of patients taking ATDs, foremost within 3 months after the start of therapy (96, 97). The onset is abrupt with high fever and severe pharyngitis (98). Agranulocytosis is dose related with respect to MMI (99), but not with PTU. Instead of a previously presumed drug-induced toxic side-effect, agranulocytosis is apparently due to immune targeting of granulocytes with subsequent alterations of the cell membrane (100-102). Specific HLA alleles in Asians and Caucasians increase susceptibility to ATD-induced agranulocytosis (103, 104). Management involves granulocyte colony-stimulating factor and antibiotics (105). PTU-induced vasculitis often occurs after years of treatment and is associated with perinuclear anticytoplasmic neutrophil antibodies (106). Hepatotoxicity occurs in 0.3% patients exposed to MMI and 0.15% of those given PTU (107, 108), while MMI and PTU are associated with noninfectious hepatitis in 0.25% and 0.08% of cases, respectively, with higher rates of hepatic failure with PTU (109, 110). Guidelines neither recommend monitoring of liver function and white blood cell count nor screening for anticytoplasmic neutrophil antibody positivity in patients on ATDs. However, timely and complete information of the patients pertaining to ATD-induced side-effects (pharyngitis, fever, jaundice, etc.) is warranted (14).

### Pregnancy

Graves hyperthyroidism affects 0.1% to 0.2% of pregnancies and increases the risk of adverse outcomes in both mother and child (78, 111-114). Optimally, hyperthyroid women should be rendered biochemically euthyroid prior to pregnancy. The managing physician is faced with challenges regarding indications and options for treatment, as well as use of ATDs. Both MMI and PTU cross the placenta with similar kinetics and may produce fetal–neonatal hypothyroidism and teratogenic effects in the embryo (77, 115-119). The rates of ATD-induced birth anomalies are 3% to 4% and 2% to 3% for MMI (aplasia cutis, esophageal and choanal atresia) and PTU (branchial fistula, renal cysts), respectively. The critical time interval for exposure of the fetus is between week 5 to 6 and week 10 (115-119).

PTU is the guideline-recommended ATD during the first trimester of pregnancy; however, if the patient was on less than 10 mg of MMI prior to pregnancy and the serum free thyroid hormone levels are within the normal range, ATDs may be stopped at week 5 and serum free T4 and free T3 controlled weekly (111). When this risk is considered high (high TSH-R-Ab, GO present, MMI dose > 10 mg/day), the lowest possible dose of PTU should be administered (50-100 mg/day) while additional levothyroxine is discouraged. In the second and third trimesters, as well as during the postpartum period and lactation, PTU is replaced by MMI to reduce potential PTU-associated liver damage (2, 14, 108, 120). Finally, TSH-R-Ab measurement is warranted in all female patients with a diagnosed autoimmune thyroid disease both prior to and during pregnancy, including those who previously had a definitive treatment (TX or RAI). The measurement of functional and specific (stimulatory or blocking) TSH-R-Abs via cell-based bioassays is clinically useful and predictive for a fetal and/or neonatal dysfunction, especially when serum TSH-R-Ab levels are higher than the 3-assay cut-off (2, 14, 44, 45, 111).

### Long-term ATD therapy

If biochemical and/or serological (TSH-R-Ab positivity) hyperthyroidism persist 18 months after the start of MMI therapy, treatment with RAI or TX is usually considered (1-3, 14). An adequate and safe alternative, which can be discussed and offered to all GD patients, is the unlimited continuation of low-dose MMI (2, 3) (Fig. 1). The same holds true for those who relapse after a previous first course of ATD. This is convincingly underlined by a recent prospective randomized clinical trial which showed a 4- to 5-fold increased cumulative incidence of hyperthyroidism recurrence in GD patients treated for an average of 19 months (±3 months) with MMI in contrast to a GD group treated for 95 months (±22 months, *P* < .001) (121). Also, a low incidence of AEs is noted on a maintenance dose (2.5-5 mg/day) of MMI (97, 122, 123), with only 1.5% of patients experiencing severe AEs (124). Furthermore, numerous studies comparing long-term outcomes (2-11 years) in GD patients with continuous ATD administration or in those randomized to either ATD or RAI demonstrated stable euthyroidism and a paucity of ATD-induced AEs (125), lower management costs, and less frequent episodes of hypothyroidism in the ATD group (126). There was also a better remission rate of 63% (127), less weight gain, and less GO deterioration compared with RAI (128).

### Children, adolescence, and elderly people

Due to its significantly increased hepatotoxic risk in young subjects, PTU is not recommended in children and adolescents (2, 14, 129). As the remission rate is markedly lower than in adults, long-term (3-5 years) MMI is the mainstay of treatment in children with GD. In contrast, definitive treatment is preferred in older patients with thyrotoxicosis-induced cardiovascular complications. Alternatively, low-dose MMI is a satisfactory treatment for older individuals with mild GD (2, 14).

### Subclinical Graves disease

Cardiovascular morbidity and mortality have been associated with endogenous subclinical GD and completely suppressed serum TSH (130-133). The rate of progression of subclinical GD to overt hyperthyroidism was 30% in the subsequent 3 years (134), especially in those patients with persistent TSH-R-Ab. Therefore, treatment of subclinical GD is recommended in GD patients in general and in those >65 years, particularly in patients with completely suppressed serum TSH levels (2). ATDs are the first treatment of choice.

### Symptomatic management and anticoagulation

Cardiac symptoms are frequently observed in patients with Graves hyperthyroidism, for example, sinus tachycardia, supraventricular arrhythmia, and atrial fibrillation in elderly people. Hence, beta-adrenergic blockade (eg, propranolol or bisoprolol) is indicated in the early stages of GD (135, 136). High doses of propranolol (160-200 mg) inhibit both thyroid hormone releases from the thyroid as well as T4 deiodination in the periphery (1, 2, 14). Anticoagulation is indicated in older patients with supraventricular arrhythmia, especially in those >65 years with atrial fibrillation (3, 135, 136).

## Radioactive Iodine

RAI treatment is indicated in (1) patients with persistent hyperthyroidism after completion of a 12- to 18-month first course of ATD therapy, (2) those with a recurrence or relapse of thyrotoxicosis, (3) patients with poor compliance on ATDs, (4) subjects with major ATD-induced side-effects, and (5) individuals who choose this approach (1-3, 14). Contraindications for RAI are (1) pregnancy and lactation, (2) GD with a nodular goiter and suspicion of thyroid cancer, (3) the presence of active GO, and (4) patients with an acknowledged high risk for the development and exacerbation of GO, for example, high stimulatory TSH-R-Ab, severe hyperthyroidism with high serum T3 levels, and smokers (1-3, 14). Indeed RAI therapy exacerbates or induces de novo GO in 15% to 20% of GD patients via a marked increase of serum stimulatory TSH-R-Ab levels (2, 55, 137). Furthermore, severe deterioration of thyrotoxicosis and/or occurrence of the thyroid storm as an endocrine emergency have been observed after RAI (138, 139). Hence, all candidates for a RAI treatment should be completely informed pertaining to the above described and acknowledged risks. The objective of the, in general, effective RAI therapy is to achieve biochemical hypothyroidism through a, frequently selected fixed, high dose of radionuclide and the subsequent destruction of the thyroid gland (140, 141). Patients should be off ATDs 1 week prior to and after RAI administration (14, 142) as ATDs negatively impact the efficacy and success of RAI. Conception is allowed 6 months at the earliest after RAI for both sexes (2, 14, 142).

Patients with differentiated thyroid cancer treated with RAI have a significantly increased risk of secondary malignancies (143). This was inconclusive for those treated with RAI for hyperthyroidism (144-146). However, recently a longitudinal study with a follow-up >25 years confirmed an increased risk of secondary cancers (breast, kidney, stomach) due to RAI treatment for hyperthyroidism. Mortality from breast (relative risk, RR 1.12) and solid cancers (RR 1.05) was significantly enhanced, while the association with solid cancer mortality increased with greater total administered RAI activity (147, 148). In a further study of over 16 000 patients treated for hyperthyroidism, a trend towards a higher risk (HR 2.32) of non-Hodgkin’s Lymphoma in RAI-treated individuals was noted (149).

## Thyroidectomy

Total TX is indicated in GD patients with (1) persistent hyperthyroidism and/or recurrence of thyrotoxicosis after the completion of a first course of ATD, (2) suspected thyroid cancer, (3) a large thyroid gland (>50-60 mL) and compressive symptoms, (4) nodular goiters and active GO, (5) concurrent hyperparathyroidism, and (6) pregnancy (second trimester) (1, 2, 14, 150). ATDs are required prior to TX especially in severe hyperthyroidism hence avoiding perioperative complications and uncontrolled thyroid dysfunction (2, 3, 14). Further, a solution containing potassium iodide given prior to TX decreases blood flow to the thyroid and intraoperative blood loss (151). Guidelines recommend that a high-volume surgeon markedly reduces the failure rate to below 1% making TX a cost-effective approach for GD (152). Potential adverse events of TX are laryngeal edema, recurrent laryngeal nerve injury, hypocalcemia, bleeding, and hypoparathyroidism (Table 1). The rate of the above complications remains low with experienced, high-volume surgeons (153-155) and the risks of transient hypocalcemia and recurrent laryngeal nerve injury are <10% and <1%, respectively (155). Calcium substitution substantially lowers the risks of perioperative hypocalcemia (156, 157). Finally, levothyroxine replacement is administered postoperatively based on a body mass index- or weight-adapted calculation (158, 159).

## Graves Orbitopathy

### Current management and its limitations

Thyroid dysfunction should be promptly restored as both hypothyroidism with elevated serum TSH as well hyperthyroidism negatively affect the clinical activity and severity of GO (3, 48). TSH directly stimulates TSH-R expressing orbital target cells to release hydrophilic mucopolysaccharides and proinflammatory cytokines (17, 160), while hyperthyroidism is associated with markedly increased serum levels of stimulatory TSH-R-Abs (18, 161). Hence, both thyroid dysfunctions exacerbate the orbital inflammatory process with local edema, congestion, and resulting exophthalmos. Adequate levothyroxine (LT4) substitution or MMI administration are warranted to reach and maintain a stable euthyroid state. RAI should be avoided in all cases of active and/or moderate to severe GO and prophylactic glucocorticoids (GCs) are recommended in GD patients treated with RAI, especially in those at high risk for developing GO, for example, high stimulatory TSH-R-Ab and smokers (2, 3, 14, 160). After confirmation of GO diagnosis, clinical disease activity, severity and presence of sight-threatening manifestations are assessed (162). Serum TSH-R-Ab levels in general and stimulatory TSH-R-Abs in particular are a reliable and highly sensitive biomarker of clinically active and severe GO (18, 42, 161, 163, 164).

The current guidelines of the European Group on Graves’ Orbitopathy (EUGOGO) and of the European Thyroid Association recommend local treatment and selenium for mild GO (165), and high-dose intravenous glucocorticoid (IVGC) pulses as standard treatment of choice (55, 166) for active and moderate to severe disease, including optic neuropathy. The disease-modifying IVGC treatment of GO is not associated with secondary adrenocortical insufficiency (167). Prior treatment of orbital disease with IVGC markedly decreases the need for successive rehabilitative surgeries (55). Inflammatory symptoms and signs as well as clinical disease activity are sensitive to IVGC with response rates averaging 70% to 80%. To improve tolerability and decrease IVGC-induced morbidity, single (>0.75 g) and cumulative doses (>8 g) of methylprednisolone, and/or a consecutive-day regimen should be avoided (55, 168, 169). In comparison, clinical severity and the corresponding signs proptosis and diplopia respond less well to IVGCs. This is why retrobulbar radiotherapy is additionally indicated in patients with disturbances of eye muscle motility (170), while orbital decompression surgery is considered in subjects with persistent active and severe GO subsequent to immunosuppressive treatment (171). Hence, current guideline-recommended approaches may still require additional surgical corrections, due to partial or insufficient improvement of severity signs, in 20% to 30% of GO cases (55, 166).

### The insulin-like growth factor 1 receptor as novel autoantigen in GO and the role of teprotumumab

The TSH-R is the primary target of orbital autoimmunity (172) as convincingly demonstrated by animal models of GO, induced by immunization with TSH-R alone (16, 173-176). Immunohistochemically analysis of human orbital tissue in GO reveals TSH-R immunoreactivity (177) and TSH-R activation impacts orbital adipogenesis (178). However, recent in vitro experiments have shown that the IGF-1R is an additional key player and a relevant autoantigen in GO pathogenesis (179). This was first hypothesized by a British group, which demonstrated the displacement of the insulin-like factor-1 (IGF-1) by GD immunoglobulins from the binding sites of the IGF-1R on cultured fibroblasts surgically obtained from GO patients (180). This novel autoantigen is expressed on the surface of orbital target cells (181) as well as on T and B cells (182) in patients with GO. The TSH-R and the IGF-1R colocalize in thyrocytes and orbital fibroblasts (183), and IGF-1R-Abs have been observed in subjects with GD and/or GO (184, 185). Subsequent to the binding of TSH-R-Abs to their ligand on TSH-R expressing cells, there is a “cross-talk” between the TSH-R and the IGF-1R leading to activation of IGF-1R-dependent downstream intracellular pathways (186-189).

Further evidence of the major role of the IGF-1R in GO has been added by in vitro testing of the novel human anti-IGF-1R monoclonal antibody (mAb) teprotumumab. Binding of teprotumumab to its ligand was able to inhibit both activation of the IGF-1R and downstream intracellular signaling by the endogenous ligands, as well as induce internalization and degradation of the IGF-1R (183). In cultured fibroblasts, this mAb suppressed the IGF-1/TSH-R-Ab-stimulated release of chemokines (190). Further, teprotumumab reduced the TSH-stimulated release of proinflammatory cytokines from isolated fibrocytes of GD patients (191), and markedly decreased the expression of both TSH-R and IGF-1R in fibrocytes (192).

Two large, consecutive, randomized, multicenter, placebo-controlled, phase II and III trials have demonstrated the efficacy and acceptable tolerability of teprotumumab in subjects with severe and active thyroid eye disease (193, 194). This drug led to a rapid and impressive improvement of all symptoms and signs of disease severity and activity during and/or after the first cycle of 8 consecutive intravenous infusions over 24 weeks (primary endpoint). In both trials, significant increases in the total score of the disease-specific quality-of-life questionnaire were also observed at week 24. Early this year, teprotumumab was approved by the Food and Drug Administration for the treatment of adult patients with severe and active GO. However, to potentially implement this promising drug in the routine management of patients with inflammatory/severe GO as well as critically evaluate its exact role and place in the guideline-recommended treatment algorithm, additional data pertaining to durability of the short-term results and long-term safety and efficacy are keenly warranted (195, 196). High costs, availability of the drug (currently commercially available in the USA only), and missing comparative trials with the current guideline-recommended standard regimen (IVGC) are additional issues of concern.

## Graves Dermopathy and Acropachy

Dermopathy and acropachy are very rare extrathyroidal manifestations of GD and occur in 1.5% to 2% and 0.3% to 0.5% of patients, respectively (197, 198). Acropachy commonly manifests as clubbing of fingers and digits (199). The pathogenesis of both of extrathyroidal manifestations is similar to GO, and practically all patients with dermopathy and/or acropachy have also moderate to severe GO (200). Local steroid therapy and occlusive dressing might be helpful for dermopathy, while no specific treatment is available for acropachy (199).

## Graves Disease in the Time of the Covid-19 Virus Pandemic

Poorly controlled hyperthyroidism increases the risk of COVID-19 infection, especially in elderly people. Uncontrolled thyrotoxicosis is associated with excess cardiovascular mortality (136, 201). Infections, for example, COVID-19, may precipitate thyroid storm in patients with poorly controlled thyroid dysfunction (202). Thus, both COVID-19 infection and an unstable hyperthyroid state markedly increase morbidity and mortality risk in general, and in elderly people in particular. Hence, each patient with Graves hyperthyroidism should continue ATD medication and should be closely monitored by the managing physician (203). Further, patients with poorly controlled diabetes and/or adrenal insufficiency, and those on high-dose immunosuppressive GCs may be more susceptible to developing COVID-19 infection, as they are with any other infections (204). The morbidity associated with those conditions could increase susceptibility, as patients with adrenal failure are more prone to develop infections (205). Concomitant electrolyte imbalances are exacerbated by infection, causing dehydration, which can propagate an adrenal crisis and a potentially fatal cytokine storm (205). Hence, GO patients on GCs may have increased susceptibility to COVID-19 as a result of the immunosuppressive effects of GCs and potential adrenal failure (204, 205). Prospective and randomized trials have shown that in contrast to oral GC therapy, IVGCs are not associated with secondary adrenal insufficiency (167, 206). As the true impact of COVID-19 in immunosuppressed patients can neither be accurately evaluated nor predicted, a cautious approach is recommended for both patients who have recently finished the IVGC treatment, and especially for those who have completed a long-term course of oral GCs for GO. These subjects should be closely monitored if they become infected by COVID-19, as they are at risk of potentially developing acute adrenal failure. Is it worth mentioning that dexamethasone improves outcomes in severe COVID-19; however, unless sight-threatening forms are present, high-doses of GC and systemic treatment should be postponed, and local as well as preventive measures offered.

## Perspectives

**Figure 2.**
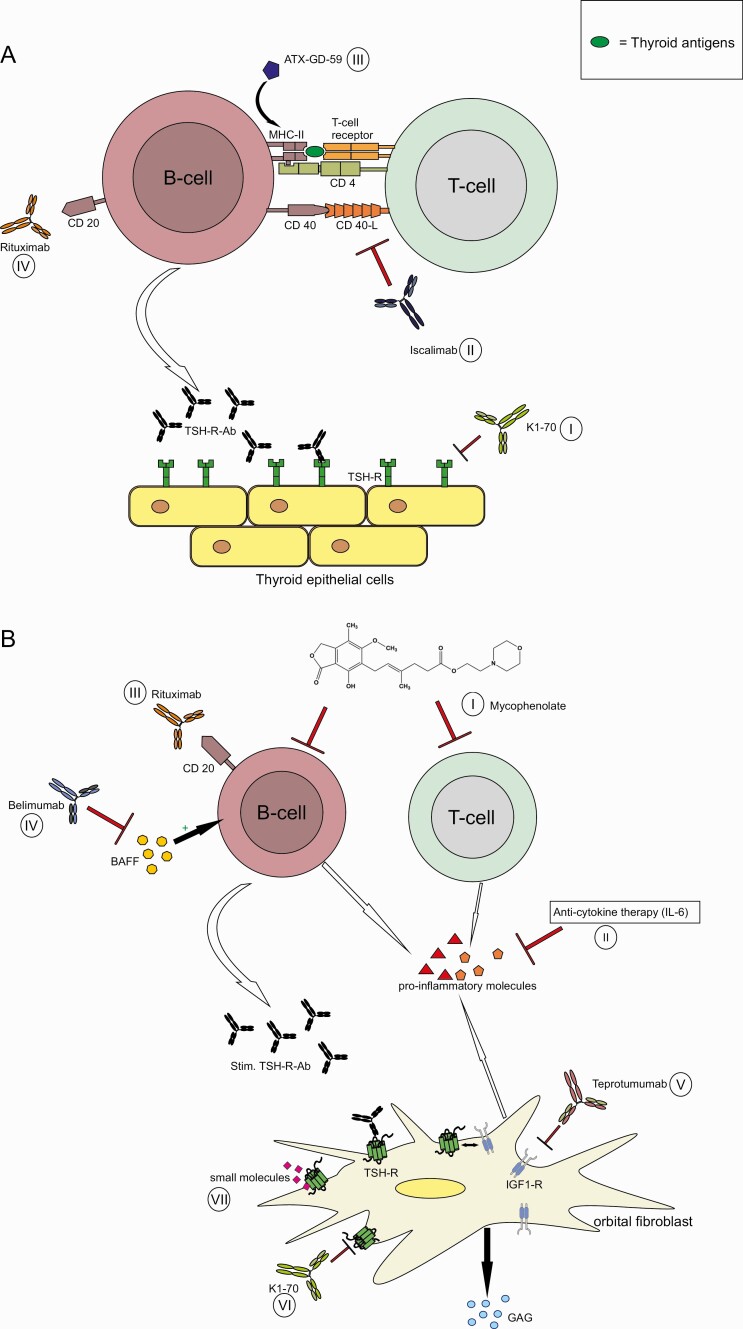
(A) Sites of action of novel treatments for Graves hyperthyroidism. Four (I-IV) mechanisms of action are represented. (I) K1-70 is a human anti-TSH-R blocking mAb. (II) Iscalimab is an anti-CD40 mAb blocking CD40-CD40 ligand (CD154) costimulatory pathway. (III) ATX-GD-59 is an “apitope” restoring immune tolerance to the TSH-R. (IV) Rituximab is an anti-CD20 Mab that inhibits B-cells and reduces autoantibody production. (B) Sites of action of novel treatments for Graves orbitopathy. Seven (I-VII) mechanisms of action are represented. (I) Mycophenolate has a dual antiproliferative effect on B and T cells. (II) Anticytokine therapies inhibit inflammatory molecules, for example, proinflammatory cytokines, chemoattractants, adhesion molecules, growth factors, etc., and include an anti-IL-6R mAb, tocilizumab, as well as anti-TNFα Ab. (III and IV) B cell targeting therapies include rituximab, an anti-CD20 mAb causing B cell depletion, and an anti-BAFF mAb (belimumab) preventing BAFF from interacting with its receptors. (V) Teprotumumab, an anti-IGF-1R mAb blocking the activation of orbital target cells (fibroblasts) with subsequent excess release of hydrophilic acidic mucopolysaccharides (GAG) and signaling of IGF-1R has been recently cleared by the FDA. (VI) K1-70 is an anti-TSH-R mAb blocking TSH-R activation by stimulatory TSH-R-Ab. (VII) Small molecules bind to the transmembrane domain of the TSH-R blocking its activation. mAb, monoclonal antibody; BAFF, B cell activating factor; CD40L, CD40 ligand; IGF-1R, insulin-like growth factor-1 receptor; IL-6R, interleukin-6 receptor; TNFα, tumor necrosis factor-α; TSH-R, thyrotropin receptor; TSH-R-Ab, thyrotropin receptor antibody; MHC class II, major histocompatibility class II molecule.

Recent in vitro and in vivo research aims to introduce and establish new treatments for the management of Graves hyperthyroidism and its associated extrathyroidal manifestations. Both the TSH-R and the IGF-1R are the primary targets for these novel, but still exploratory, approaches. Other targets include either molecules relevant to the pathogenesis of GD/GO that play an important role within the immunological synapse and are expressed on the surface of B cells (eg, CD40 and CD20), or important proinflammatory cytokine receptors, for example, IL-6R (Fig. 2A and Fig. 2B) or tumor necrosis factor (TNF)α. In line with this, 2 TNFα blockers, etanercept (207) and adalimumab (208), were tested with modest benefit. In contrast, far better results were observed with mycophenolate, an immunosuppressive drug with dual antiproliferative effects on T and B cells as well as on orbital target cells (209). Two randomized controlled trials encompassing more than 300 patients with active and severe GO demonstrated the safety and good tolerability of mycophenolate, as well as beneficial effects of the drug on eye symptoms and signs, clinical disease severity (proptosis, motility disturbances, and/or diplopia), and clinical activity (orbital inflammation) (210, 211). This clinical benefit was convincingly accompanied by a markedly improved disease-specific quality of life. Overall, the risk–benefit ratio of mycophenolate treatment in GO was highly favorable (212, 213). Hence, mycophenolate is recommended as an efficacious, safe, and well-tolerated add-on drug to IVGCs in active/severe GO.

However, the future causal treatment of GD/GO belongs to mAbs or small molecules that can inhibit activation/hypertrophy of thyrocytes and orbital target cells. In detail, the disease-specific and most effective way to inhibit the increased release of free thyroid hormones induced by GD is to administer a TSH-R antagonist able to block the TSH-R and the stimulatory effect of the TSH-R-Abs. In the United Kingdom, clinical trials testing the efficacy and safety of a monoclonal human antibody (K1-70) targeting and blocking TSH-R activation by stimulatory TSH-R-Abs are ongoing (214, 215). Alternatively, small molecules which bind to the transmembrane domain of the TSH-R subsequently blocking its activation were recently introduced. A novel, selective TSH-R small-molecule antagonist (S37a) was reported to inhibit both TSH-R activation by TSH itself and activation by M22, a stimulating anti-TSH-R human mAb (216). Further, the clinical effect of a combination of different TSH-R peptides called ATX-GD-59 (217), was assessed in untreated subjects with Graves hyperthyroidism. ATX-GD-59 is an “apitope,” restoring immune tolerance to the TSH-R. This “apitope” is able to both suppress the immune response against the TSH-R and generate suppressor, regulatory T cells. Seven of 10 treated GD patients showed a complete or partial response to ATX-GD-59 (218).

The CD40 molecule is abundantly expressed by thyrocytes and orbital target cells and CD40–CD154 interaction modulates humoral immunity within the immunological synapse (219, 220). Iscalimab, an anti-CD40 mAb and a potent inhibitor of the CD40-CD40 ligand (CD154) costimulatory pathway, was administered to patients with untreated GD. Iscalimab was very well tolerated, with half of the subjects responding to the drug (221). In comparison, B cell targeting therapies include rituximab, an anti-CD20 humanized mAb causing B cell depletion, and an anti-BAFF mAb (belimumab) preventing BAFF from interacting with its receptors. Rituximab has been tested in GD leading to a decline of TSH-R-Abs (222). When administered to GO patients, grossly conflicting results were registered (223, 224). High costs, low efficacy, and potential drug–related side effects (orbital edema and optic neuropathy) are major hurdles for the generalized use of rituximab in GO (225). Finally, when tested in an open-label study (226), a humanized recombinant IL6-R mAb (Tocilizumab) positively improved the clinical course of the disease and extraocular muscle motility in patients with steroid-resistant GO. However, a subsequent tocilizumab placebo-controlled randomized trial in GO patients only partially reproduced the earlier reported impressive findings (227).

In conclusion, further controlled randomized studies are warranted to demonstrate the efficacy, safety, and clinical role of the above new compounds for GD and GO.

## Data Availability

Data sharing is not applicable to this article as no datasets were generated or analyzed during the current study.

## Abbreviations

Ab: autoantibodies
AE: adverse event
ATD: thionamide antithyroid drug
CBM: carbimazole
GD: Graves disease
GO: Graves orbitopathy
GC: glucocorticoid
IGF-1R: insulin-like growth factor-1 receptor
IVGC: intravenous glucocorticoid
mAb: monoclonal antibody
MMI: methimazole
PTU: propylthiouracil
RAI: radioactive iodine
RR: relative risk
T3: 3,5,3′-triiodothyronine
T4: thyroxine
TNF: tumor necrosis factor
TSAb: stimulatory TSH-R-Ab
TSH: thyrotropin
TSH-R-Ab: thyrotropin receptor antibody
TX: thyroidectomy

## Additional Information

***Disclosure Summary:*** The JGU Medical Center has received research-associated funding from the JGU Medical School, AdvanceCor, Germany, Apitope, United Kingdom, Horizon therapeutics, USA, Immunovant, USA, Merck, Germany, Novartis, USA, Quidel, USA, River Vision, USA, and Synthon, The Netherlands. G.J.K. consults for Immunovant, USA, Merck, Germany, Novartis, Switzerland, and Quidel, USA

## References

[1] Bahn Chair RS, Burch HB, Cooper DS, et al.; American Thyroid Association; American Association of Clinical Endocrinologists. Hyperthyroidism and other causes of thyrotoxicosis: management guidelines of the American Thyroid Association and American Association of Clinical Endocrinologists. Thyroid. 2011;21(6):593-646. 21510801 10.1089/thy.2010.0417

[2] Kahaly GJ, Bartalena L, Hegedüs L, Leenhardt L, Poppe K, Pearce SH. 2018 European Thyroid Association Guideline for the management of Graves’ hyperthyroidism. Eur Thyroid J. 2018;7(4):167-186. 30283735 10.1159/000490384 PMC6140607

[3] Bartalena L . Diagnosis and management of Graves disease: a global overview. Nat Rev Endocrinol. 2013;9(12):724-734. 24126481 10.1038/nrendo.2013.193

[4] Nyström HF, Jansson S, Berg G. Incidence rate and clinical features of hyperthyroidism in a long-term iodine sufficient area of Sweden (Gothenburg) 2003-2005. Clin Endocrinol (Oxf). 2013;78(5):768-776. 23421407 10.1111/cen.12060

[5] McLeod DS, Caturegli P, Cooper DS, Matos PG, Hutfless S. Variation in rates of autoimmune thyroid disease by race/ethnicity in US military personnel. JAMA. 2014;311(15):1563-1565. 24737370 10.1001/jama.2013.285606

[6] Dittmar M, Libich C, Brenzel T, Kahaly GJ. Increased familial clustering of autoimmune thyroid diseases. Horm Metab Res. 2011;43(3):200-204. 21287436 10.1055/s-0031-1271619

[7] Frommer L, Kahaly GJ. Autoimmune polyendocrinopathy. J Clin Endocrinol Metab. 2019;104(10):4769-4782. 31127843 10.1210/jc.2019-00602

[8] Kahaly GJ, Frommer L. Polyglandular autoimmune syndromes. J Endocrinol Invest. 2018;41(1):91-98. 28819917 10.1007/s40618-017-0740-9

[9] Kahaly GJ, Frommer L. Autoimmune polyglandular diseases. Best Pract Res Clin Endocrinol Metab. 2019;33(6):101344. 31606344 10.1016/j.beem.2019.101344

[10] Brix TH, Kyvik KO, Christensen K, Hegedüs L. Evidence for a major role of heredity in Graves’ disease: a population-based study of two Danish twin cohorts. J Clin Endocrinol Metab. 2001;86(2):930-934. 11158069 10.1210/jcem.86.2.7242

[11] Strieder TG, Prummel MF, Tijssen JG, Endert E, Wiersinga WM. Risk factors for and prevalence of thyroid disorders in a cross-sectional study among healthy female relatives of patients with autoimmune thyroid disease. Clin Endocrinol (Oxf). 2003;59(3):396-401. 12919165 10.1046/j.1365-2265.2003.01862.x

[12] Laurberg P, Pedersen KM, Vestergaard H, Sigurdsson G. High incidence of multinodular toxic goitre in the elderly population in a low iodine intake area vs. high incidence of Graves’ disease in the young in a high iodine intake area: comparative surveys of thyrotoxicosis epidemiology in East-Jutland Denmark and Iceland. J Intern Med. 1991;229(5):415-420. 2040867 10.1111/j.1365-2796.1991.tb00368.x

[13] Brix TH, Hansen PS, Kyvik KO, Hegedüs L. Cigarette smoking and risk of clinically overt thyroid disease: a population-based twin case-control study. Arch Intern Med. 2000;160(5):661-666. 10724051 10.1001/archinte.160.5.661

[14] Ross DS, Burch HB, Cooper DS, et al. 2016 American Thyroid Association Guidelines for diagnosis and management of hyperthyroidism and other causes of thyrotoxicosis. Thyroid. 2016;26(10):1343-1421. 27521067 10.1089/thy.2016.0229

[15] Diana T, Daiber A, Oelze M, et al. Stimulatory TSH-receptor antibodies and oxidative stress in Graves disease. J Clin Endocrinol Metab. 2018;103(10):3668-3677. 30099546 10.1210/jc.2018-00509 PMC6179174

[16] Diana T, Olivo PD, Chang YH, Wüster C, Kanitz M, Kahaly GJ. Comparison of a novel homogeneous cyclic amp assay and a luciferase assay for measuring stimulating thyrotropin-receptor autoantibodies. Eur Thyroid J. 2020;9(2):67-72. 32257955 10.1159/000504509 PMC7109431

[17] Davies TF, Andersen S, Latif R, et al. Graves’ disease. Nat Rev Dis Primers. 2020;6(1):52. 32616746 10.1038/s41572-020-0184-y

[18] Kahaly GJ, Diana T, Olivo PD. TSH receptor antibodies: relevance & utility. Endocr Pract. 2020;26(1):97-106. 32022598 10.4158/EP-2019-0363

[19] Grebe SK, Kahaly GJ. Laboratory testing in hyperthyroidism. Am J Med. 2012;125(9):S2. 10.1016/j.amjmed.2012.05.013 22938936

[20] de los Santos ET, Starich GH, Mazzaferri EL. Sensitivity, specificity, and cost-effectiveness of the sensitive thyrotropin assay in the diagnosis of thyroid disease in ambulatory patients. Arch Intern Med. 1989;149(3):526-532. 2493228 10.1001/archinte.149.3.526

[21] Spencer CA, LoPresti JS, Patel A, et al. Applications of a new chemiluminometric thyrotropin assay to subnormal measurement. J Clin Endocrinol Metab. 1990;70(2):453-460. 2105333 10.1210/jcem-70-2-453

[22] Bartalena L, Burch HB, Burman KD, Kahaly GJ. A 2013 European survey of clinical practice patterns in the management of Graves’ disease. Clin Endocrinol (Oxf). 2016;84(1):115-120. 25581877 10.1111/cen.12688

[23] Kahaly GJ, Olivo PD. Graves’ disease. N Engl J Med. 2017;376(2):184. 10.1056/NEJMc1614624 28079341

[24] Kahaly GJ, Diana T. TSH receptor antibody functionality and nomenclature. Front Endocrinol (Lausanne). 2017;8:28. 28261158 10.3389/fendo.2017.00028 PMC5309226

[25] Tozzoli R, Bagnasco M, Giavarina D, Bizzaro N. TSH receptor autoantibody immunoassay in patients with Graves’ disease: improvement of diagnostic accuracy over different generations of methods. Systematic review and meta-analysis. Autoimmun Rev. 2012;12(2):107-113. 22776786 10.1016/j.autrev.2012.07.003

[26] Lytton SD, Li Y, Olivo PD, Kohn LD, Kahaly GJ. Novel chimeric thyroid-stimulating hormone-receptor bioassay for thyroid-stimulating immunoglobulins. Clin Exp Immunol. 2010;162(3):438-446. 21070207 10.1111/j.1365-2249.2010.04266.x PMC3026547

[27] Leschik JJ, Diana T, Olivo PD, et al. Analytical performance and clinical utility of a bioassay for thyroid-stimulating immunoglobulins. Am J Clin Pathol. 2013;139(2):192-200. 23355204 10.1309/AJCPZUT7CNUEU7OP

[28] Li Y, Kim J, Diana T, Klasen R, Olivo PD, Kahaly GJ. A novel bioassay for anti-thyrotrophin receptor autoantibodies detects both thyroid-blocking and stimulating activity. Clin Exp Immunol. 2013;173(3):390-397. 23647395 10.1111/cei.12129 PMC3949626

[29] Araki N, Iida M, Amino N, et al. Rapid bioassay for detection of thyroid-stimulating antibodies using cyclic adenosine monophosphate-gated calcium channel and aequorin. Eur Thyroid J. 2015;4(1):14-19. 25960957 10.1159/000371740 PMC4404900

[30] Kahaly GJ . Bioassays for TSH receptor antibodies: Quo Vadis? Eur Thyroid J. 2015;4(1):3-5. 25960955 10.1159/000375445 PMC4404890

[31] Diana T, Kanitz M, Lehmann M, Li Y, Olivo PD, Kahaly GJ. Standardization of a bioassay for thyrotropin receptor stimulating autoantibodies. Thyroid. 2015;25(2):169-175. 25317659 10.1089/thy.2014.0346

[32] Diana T, Li Y, Olivo PD, et al. Analytical performance and validation of a bioassay for thyroid-blocking antibodies. Thyroid. 2016;26(5):734-740. 26956921 10.1089/thy.2015.0447

[33] Diana T, Krause J, Olivo PD, et al. Prevalence and clinical relevance of thyroid stimulating hormone receptor-blocking antibodies in autoimmune thyroid disease. Clin Exp Immunol. 2017;189(3):304-309. 28439882 10.1111/cei.12980 PMC5543506

[34] Diana T, Wüster C, Kanitz M, Kahaly GJ. Highly variable sensitivity of five binding and two bio-assays for TSH-receptor antibodies. J Endocrinol Invest. 2016;39(10):1159-1165. 27197966 10.1007/s40618-016-0478-9

[35] Diana T, Wüster C, Olivo PD, et al. Performance and specificity of 6 immunoassays for TSH receptor antibodies: a multicenter study. Eur Thyroid J. 2017;6(5):243-249. 29071236 10.1159/000478522 PMC5649260

[36] Allelein S, Diana T, Ehlers M, et al. Comparison of a bridge immunoassay with two bioassays for thyrotropin receptor antibody detection and differentiation. Horm Metab Res. 2019;51(6):341-346. 31207654 10.1055/a-0914-0535

[37] Lytton SD, Ponto KA, Kanitz M, Matheis N, Kohn LD, Kahaly GJ. A novel thyroid stimulating immunoglobulin bioassay is a functional indicator of activity and severity of Graves’ orbitopathy. J Clin Endocrinol Metab. 2010;95(5):2123-2131. 20237164 10.1210/jc.2009-2470

[38] Ponto KA, Kanitz M, Olivo PD, Pitz S, Pfeiffer N, Kahaly GJ. Clinical relevance of thyroid-stimulating immunoglobulins in Graves’ ophthalmopathy. Ophthalmology. 2011;118(11):2279-2285. 21684605 10.1016/j.ophtha.2011.03.030

[39] Diana T, Brown RS, Bossowski A, et al. Clinical relevance of thyroid-stimulating autoantibodies in pediatric Graves’ disease-a multicenter study. J Clin Endocrinol Metab. 2014;99(5):1648-1655. 24517152 10.1210/jc.2013-4026

[40] Ponto KA, Diana T, Binder H, et al. Thyroid-stimulating immunoglobulins indicate the onset of dysthyroid optic neuropathy. J Endocrinol Invest. 2015;38(7):769-777. 25736545 10.1007/s40618-015-0254-2

[41] Kampmann E, Diana T, Kanitz M, Hoppe D, Kahaly GJ. Thyroid stimulating but not blocking autoantibodies are highly prevalent in severe and active thyroid-associated orbitopathy: a prospective study. Int J Endocrinol. 2015;2015(1):678194. 26221139 10.1155/2015/678194 PMC4499387

[42] Kahaly GJ, Diana T, Glang J, Kanitz M, Pitz S, König J. Thyroid stimulating antibodies are highly prevalent in Hashimoto’s thyroiditis and associated orbitopathy. J Clin Endocrinol Metab. 2016;101(5):1998-2004. 26964732 10.1210/jc.2016-1220

[43] Stożek K, Bossowski A, Ziora K, et al. Functional TSH receptor antibodies in children with autoimmune thyroid diseases. Autoimmunity. 2018;51(2):62-68. 29372654 10.1080/08916934.2018.1431776

[44] Kiefer FW, Klebermass-Schrehof K, Steiner M, et al. Fetal/neonatal thyrotoxicosis in a newborn from a hypothyroid woman with Hashimoto thyroiditis. J Clin Endocrinol Metab. 2017;102(1):6-9. 27813690 10.1210/jc.2016-2999

[45] Decallonne B, Martens PJ, Van den Bruel A, Vanhole C, Kahaly GJ. Graves Disease with thyroid-stimulating hormone receptor-blocking autoantibodies during pregnancy. Ann Intern Med. 2020;172(11):767-769. 32203974 10.7326/L19-0818

[46] Mestman J . Fetal hyperthyroidism resulted from TSI in a mother with Hashimoto’s hypothyroidism. Clin Thyroidol. 2017;29(1):32-34.

[47] McKee A, Peyerl F. TSI assay utilization: impact on costs of Graves’ hyperthyroidism diagnosis. Am J Manag Care. 2012;18(1):e1-14. 22435785

[48] Kahaly GJ, Bartalena L, Hegedüs L. The American Thyroid Association/American Association of Clinical Endocrinologists guidelines for hyperthyroidism and other causes of thyrotoxicosis: a European perspective. Thyroid. 2011;21(6):585-591. 21663420 10.1089/thy.2011.2106.ed3

[49] Vitti P, Rago T, Mancusi F, et al. Thyroid hypoechogenic pattern at ultrasonography as a tool for predicting recurrence of hyperthyroidism after medical treatment in patients with Graves’ disease. Acta Endocrinol (Copenh). 1992;126(2):128-131. 1543017 10.1530/acta.0.1260128

[50] Hegedüs L . Thyroid ultrasound. Endocrinol Metab Clin North Am. 2001;30(2):339-360, viii. 11444166 10.1016/s0889-8529(05)70190-0

[51] Erdoğan MF, Anil C, Cesur M, Başkal N, Erdoğan G. Color flow Doppler sonography for the etiologic diagnosis of hyperthyroidism. Thyroid. 2007;17(3):223-228. 17381355 10.1089/thy.2006.0104

[52] Ralls PW, Mayekawa DS, Lee KP, et al. Color-flow Doppler sonography in Graves disease: “thyroid inferno”. AJR Am J Roentgenol. 1988;150(4):781-784. 3279732 10.2214/ajr.150.4.781

[53] Burch HB, Burman KD, Cooper DS. A 2011 survey of clinical practice patterns in the management of Graves’ disease. J Clin Endocrinol Metab. 2012;97(12):4549-4558. 23043191 10.1210/jc.2012-2802

[54] Brito JP, Schilz S, Singh Ospina N, et al. Antithyroid drugs-the most common treatment for Graves’ disease in the United States: a nationwide population-based study. Thyroid. 2016;26(8):1144-1145. 27267495 10.1089/thy.2016.0222

[55] Bartalena L, Baldeschi L, Boboridis K, et al.; European Group on Graves’ Orbitopathy (EUGOGO). The 2016 European Thyroid Association/European Group on Graves’ Orbitopathy Guidelines for the management of Graves’ orbitopathy. Eur Thyroid J. 2016;5(1):9-26. 27099835 10.1159/000443828 PMC4836120

[56] Ma C, Xie J, Wang H, Li J, Chen S. Radioiodine therapy versus antithyroid medications for Graves’ disease. Cochrane Database Syst Rev. 2016;2:CD010094. 26891370 10.1002/14651858.CD010094.pub2 PMC10517434

[57] Fanning E, Inder WJ, Mackenzie E. Radioiodine treatment for graves’ disease: a 10-year Australian cohort study. BMC Endocr Disord. 2018;18(1):94. 30541519 10.1186/s12902-018-0322-7 PMC6292026

[58] Bartalena L, Marcocci C, Bogazzi F, et al. Relation between therapy for hyperthyroidism and the course of Graves’ ophthalmopathy. N Engl J Med. 1998;338(2):73-78. 9420337 10.1056/NEJM199801083380201

[59] Tallstedt L, Lundell G, Tørring O, et al. Occurrence of ophthalmopathy after treatment for Graves’ hyperthyroidism. The Thyroid Study Group. N Engl J Med. 1992;326(26):1733-1738. 1489388 10.1056/NEJM199206253262603

[60] Träisk F, Tallstedt L, Abraham-Nordling M, et al.; Thyroid Study Group of TT 96. Thyroid-associated ophthalmopathy after treatment for Graves’ hyperthyroidism with antithyroid drugs or iodine-131. J Clin Endocrinol Metab. 2009;94(10):3700-3707. 19723755 10.1210/jc.2009-0747

[61] Li HX, Xiang N, Hu WK, Jiao XL. Relation between therapy options for Graves’ disease and the course of Graves’ ophthalmopathy: a systematic review and meta-analysis. J Endocrinol Invest. 2016;39(11):1225-1233. 27220843 10.1007/s40618-016-0484-y

[62] Laurberg P, Wallin G, Tallstedt L, Abraham-Nordling M, Lundell G, Tørring O. TSH-receptor autoimmunity in Graves’ disease after therapy with anti-thyroid drugs, surgery, or radioiodine: a 5-year prospective randomized study. Eur J Endocrinol. 2008;158(1):69-75. 18166819 10.1530/EJE-07-0450

[63] Davidson B, Soodak M, Neary JT, et al. The irreversible inactivation of thyroid peroxidase by methylmercaptoimidazole, thiouracil, and propylthiouracil in vitro and its relationship to in vivo findings. Endocrinology. 1978;103(3):871-882. 744122 10.1210/endo-103-3-871

[64] Taurog A, Riesco G, Larsen PR. Formation of 3,3’-diiodothyronine and 3’,5’,3-triiodothyronine (reverse T3) in thyroid glands of rats and in enzymatically iodinated thyroglobulin. Endocrinology. 1976;99(1):281-290. 939197 10.1210/endo-99-1-281

[65] Humar M, Dohrmann H, Stein P, et al. Thionamides inhibit the transcription factor nuclear factor-kappaB by suppression of Rac1 and inhibitor of kappaB kinase alpha. J Pharmacol Exp Ther. 2008;324(3):1037-1044. 18055877 10.1124/jpet.107.132407

[66] Tötterman TH, Karlsson FA, Bengtsson M, Mendel-Hartvig I. Induction of circulating activated suppressor-like T cells by methimazole therapy for Graves’ disease. N Engl J Med. 1987;316(1):15-22. 2946953 10.1056/NEJM198701013160104

[67] Zantut-Wittmann DE, Tambascia MA, da Silva Trevisan MA, Pinto GA, Vassallo J. Antithyroid drugs inhibit in vivo HLA-DR expression in thyroid follicular cells in Graves’ disease. Thyroid. 2001;11(6):575-580. 11442005 10.1089/105072501750302886

[68] Imamura M, Aoki N, Saito T, et al. Inhibitory effects of antithyroid drugs on oxygen radical formation in human neutrophils. Acta Endocrinol (Copenh). 1986;112(2):210-216. 2426912 10.1530/acta.0.1120210

[69] Weetman AP, Holt ME, Campbell AK, Hall R, McGregor AM. Methimazole and generation of oxygen radicals by monocytes: potential role in immunosuppression. Br Med J (Clin Res Ed). 1984;288(6416):518-520. 10.1136/bmj.288.6416.518 PMC1444568 6421361

[70] Kim KA, von Zastrow M. Old drugs learn new tricks: insights from mammalian trace amine receptors. Mol Pharmacol. 2001;60(6):1165-1167. 11723221 10.1124/mol.60.6.1165

[71] Jansson R, Dahlberg PA, Johansson H, Lindström B. Intrathyroidal concentrations of methimazole in patients with Graves’ disease. J Clin Endocrinol Metab. 1983;57(1):129-132. 6687892 10.1210/jcem-57-1-129

[72] Visser TJ, van Overmeeren-Kaptein E. Study on the enzymatic 5’-deiodination of 3’,5’-diiodothyronine using a radioimmunoassay for 3’-iodothyronine. Biochim Biophys Acta. 1980;631(2):246-252. 7407247 10.1016/0304-4165(80)90299-8

[73] Kuiper GG, Kester MH, Peeters RP, Visser TJ. Biochemical mechanisms of thyroid hormone deiodination. Thyroid. 2005;15(8):787-798. 16131322 10.1089/thy.2005.15.787

[74] Cooper DS . Antithyroid drugs. N Engl J Med. 2005;352(9):905-917. 15745981 10.1056/NEJMra042972

[75] Cooper DS . Antithyroid drugs in the management of patients with Graves’ disease: an evidence-based approach to therapeutic controversies. J Clin Endocrinol Metab. 2003;88(8):3474-3481. 12915620 10.1210/jc.2003-030185

[76] Jansson R, Dahlberg PA, Lindström B. Comparative bioavailability of carbimazole and methimazole. Int J Clin Pharmacol Ther Toxicol. 1983;21(10):505-510. 6642787

[77] Andersen SL, Olsen J, Wu CS, Laurberg P. Birth defects after early pregnancy use of antithyroid drugs: a Danish nationwide study. J Clin Endocrinol Metab. 2013;98(11):4373-4381. 24151287 10.1210/jc.2013-2831

[78] Cooper DS, Laurberg P. Hyperthyroidism in pregnancy. Lancet Diabetes Endocrinol. 2013;1(3):238-249. 24622372 10.1016/S2213-8587(13)70086-X

[79] Burch HB, Wartofsky L. Life-threatening thyrotoxicosis. Thyroid storm. Endocrinol Metab Clin North Am. 1993;22(2):263-277. 8325286

[80] Abraham P, Avenell A, McGeoch SC, et al. Antithyroid drug regimen for treating Graves’ hyperthyroidism. Cochrane Database Syst Rev. 2010;1:CD003420. 20091544 10.1002/14651858.CD003420.pub4 PMC6599817

[81] McIver B, Rae P, Beckett G, Wilkinson E, Gold A, Toft A. Lack of effect of thyroxine in patients with Graves’ hyperthyroidism who are treated with an antithyroid drug. N Engl J Med. 1996;334(4):220-224. 8531998 10.1056/NEJM199601253340403

[82] Pujol P, Osman A, Grabar S, et al. TSH suppression combined with carbimazole for Graves’ disease: effect on remission and relapse rates. Clin Endocrinol (Oxf). 1998;48(5):635-640. 9666876 10.1046/j.1365-2265.1998.00466.x

[83] Rittmaster RS, Abbott EC, Douglas R, et al. Effect of methimazole, with or without L-thyroxine, on remission rates in Graves’ disease. J Clin Endocrinol Metab. 1998;83(3):814-818. 9506733 10.1210/jcem.83.3.4613

[84] Glinoer D, de Nayer P, Bex M; Belgian Collaborative Study Group on Graves’ Disease. Effects of l-thyroxine administration, TSH-receptor antibodies and smoking on the risk of recurrence in Graves’ hyperthyroidism treated with antithyroid drugs: a double-blind prospective randomized study. Eur J Endocrinol. 2001;144(5):475-483. 11331213 10.1530/eje.0.1440475

[85] Okamoto Y, Tanigawa S, Ishikawa K, Hamada N. TSH receptor antibody measurements and prediction of remission in Graves’ disease patients treated with minimum maintenance doses of antithyroid drugs. Endocr J. 2006;53(4):467-472. 16820704 10.1507/endocrj.k05-121

[86] Vitti P, Rago T, Chiovato L, et al. Clinical features of patients with Graves’ disease undergoing remission after antithyroid drug treatment. Thyroid. 1997;7(3):369-375. 9226205 10.1089/thy.1997.7.369

[87] Kimball LE, Kulinskaya E, Brown B, Johnston C, Farid NR. Does smoking increase relapse rates in Graves’ disease? J Endocrinol Invest. 2002;25(2):152-157. 11929086 10.1007/BF03343979

[88] Rotondi M, Cappelli C, Pirali B, et al. The effect of pregnancy on subsequent relapse from Graves’ disease after a successful course of antithyroid drug therapy. J Clin Endocrinol Metab. 2008;93(10):3985-3988. 18664537 10.1210/jc.2008-0966

[89] Azizi F, Malboosbaf R. Long-term antithyroid drug treatment: a systematic review and meta-analysis. Thyroid. 2017;27(10):1223-1231. 28699478 10.1089/thy.2016.0652

[90] Vos XG, Endert E, Zwinderman AH, Tijssen JG, Wiersinga WM. Predicting the risk of recurrence before the start of antithyroid drug therapy in patients with Graves’ hyperthyroidism. J Clin Endocrinol Metab. 2016;101(4):1381-1389. 26863422 10.1210/jc.2015-3644

[91] Masiello E, Veronesi G, Gallo D, et al. Antithyroid drug treatment for Graves’ disease: baseline predictive models of relapse after treatment for a patient-tailored management. J Endocrinol Invest. 2018;41(12):1425-1432. 29946800 10.1007/s40618-018-0918-9

[92] Nakamura H, Noh JY, Itoh K, Fukata S, Miyauchi A, Hamada N. Comparison of methimazole and propylthiouracil in patients with hyperthyroidism caused by Graves’ disease. J Clin Endocrinol Metab. 2007;92(6):2157-2162. 17389704 10.1210/jc.2006-2135

[93] Brix TH, Lund LC, Henriksen DP, et al. Methimazole and risk of acute pancreatitis. Lancet Diabetes Endocrinol. 2020;8(3):187-189. 32035032 10.1016/S2213-8587(20)30025-5

[94] Cooper D . The association between thionamides and acute pancreatitis. Clin Thyroidol 2020;32(7):327-329.

[95] Guo JY, Chang CL, Chen CC. Association between thionamides and acute pancreatitis: a case-control study. [Published online on June 10, 2020]. Thyroid. 2020. Doi: 10.1089/thy.2019.0589 PMC7692926 32380933

[96] Tajiri J, Noguchi S, Murakami T, Murakami N. Antithyroid drug-induced agranulocytosis. The usefulness of routine white blood cell count monitoring. Arch Intern Med. 1990;150(3):621-624. 2310281 10.1001/archinte.150.3.621

[97] Nakamura H, Miyauchi A, Miyawaki N, Imagawa J. Analysis of 754 cases of antithyroid drug-induced agranulocytosis over 30 years in Japan. J Clin Endocrinol Metab. 2013;98(12):4776-4783. 24057289 10.1210/jc.2013-2569

[98] Sheng WH, Hung CC, Chen YC, et al. Antithyroid-drug-induced agranulocytosis complicated by life-threatening infections. QJM. 1999;92(8):455-461. 10627862 10.1093/qjmed/92.8.455

[99] Takata K, Kubota S, Fukata S, et al. Methimazole-induced agranulocytosis in patients with Graves’ disease is more frequent with an initial dose of 30 mg daily than with 15 mg daily. Thyroid. 2009;19(6):559-563. 19445623 10.1089/thy.2008.0364

[100] Fibbe WE, Claas FH, Van der Star-Dijkstra W, Schaafsma MR, Meyboom RH, Falkenburg JH. Agranulocytosis induced by propylthiouracil: evidence of a drug dependent antibody reacting with granulocytes, monocytes and haematopoietic progenitor cells. Br J Haematol. 1986;64(2):363-373. 3778829 10.1111/j.1365-2141.1986.tb04130.x

[101] Akamizu T, Ozaki S, Hiratani H, et al. Drug-induced neutropenia associated with anti-neutrophil cytoplasmic antibodies (ANCA): possible involvement of complement in granulocyte cytotoxicity. Clin Exp Immunol. 2002;127(1):92-98. 11882038 10.1046/j.1365-2249.2002.01720.x PMC1906299

[102] Johnston A, Uetrecht J. Current understanding of the mechanisms of idiosyncratic drug-induced agranulocytosis. Expert Opin Drug Metab Toxicol. 2015;11(2):243-257. 25424130 10.1517/17425255.2015.985649

[103] Chen PL, Shih SR, Wang PW, et al. Genetic determinants of antithyroid drug-induced agranulocytosis by human leukocyte antigen genotyping and genome-wide association study. Nat Commun. 2015;6(7633):7633. 26151496 10.1038/ncomms8633 PMC4506516

[104] Hallberg P, Eriksson N, Ibañez L, et al.; EuDAC collaborators. Genetic variants associated with antithyroid drug-induced agranulocytosis: a genome-wide association study in a European population. Lancet Diabetes Endocrinol. 2016;4(6):507-516. 27157822 10.1016/S2213-8587(16)00113-3

[105] Andrès E, Zimmer J, Mecili M, Weitten T, Alt M, Maloisel F. Clinical presentation and management of drug-induced agranulocytosis. Expert Rev Hematol. 2011;4(2):143-151. 21495924 10.1586/ehm.11.12

[106] Balavoine AS, Glinoer D, Dubucquoi S, Wémeau JL. Antineutrophil cytoplasmic antibody-positive small-vessel vasculitis associated with antithyroid drug therapy: how significant is the clinical problem? Thyroid. 2015;25(12):1273-1281. 26414658 10.1089/thy.2014.0603

[107] Woeber KA . Methimazole-induced hepatotoxicity. Endocr Pract. 2002;8(3):222-224. 12467281 10.4158/EP.8.3.222

[108] Yang J, Li LF, Xu Q, et al. Analysis of 90 cases of antithyroid drug-induced severe hepatotoxicity over 13 years in China. Thyroid. 2015;25(3):278-283. 25384184 10.1089/thy.2014.0350

[109] Wang MT, Lee WJ, Huang TY, Chu CL, Hsieh CH. Antithyroid drug-related hepatotoxicity in hyperthyroidism patients: a population-based cohort study. Br J Clin Pharmacol. 2014;78(3):619-629. 25279406 10.1111/bcp.12336 PMC4243912

[110] Huang MJ, Liaw YF. Clinical associations between thyroid and liver diseases. J Gastroenterol Hepatol. 1995;10(3):344-350. 7548816 10.1111/j.1440-1746.1995.tb01106.x

[111] Alexander EK, Pearce EN, Brent GA, et al. 2017 Guidelines of the American Thyroid Association for the diagnosis and management of thyroid disease during pregnancy and the postpartum. Thyroid. 2017;27(3):315-389. 28056690 10.1089/thy.2016.0457

[112] Momotani N, Ito K, Hamada N, Ban Y, Nishikawa Y, Mimura T. Maternal hyperthyroidism and congenital malformation in the offspring. Clin Endocrinol (Oxf). 1984;20(6):695-700. 6467634 10.1111/j.1365-2265.1984.tb00119.x

[113] Kriplani A, Buckshee K, Bhargava VL, Takkar D, Ammini AC. Maternal and perinatal outcome in thyrotoxicosis complicating pregnancy. Eur J Obstet Gynecol Reprod Biol. 1994;54(3):159-163. 7523202 10.1016/0028-2243(94)90276-3

[114] Andersen SL, Andersen S, Vestergaard P, Olsen J. Maternal thyroid function in early pregnancy and child neurodevelopmental disorders: a Danish nationwide case-cohort study. Thyroid. 2018;28(4):537-546. 29584590 10.1089/thy.2017.0425

[115] Laurberg P, Andersen SL. Therapy of endocrine disease: antithyroid drug use in early pregnancy and birth defects: time windows of relative safety and high risk? Eur J Endocrinol. 2014;171(1):R13-R20. 24662319 10.1530/EJE-14-0135

[116] Clementi M, Di Gianantonio E, Pelo E, Mammi I, Basile RT, Tenconi R. Methimazole embryopathy: delineation of the phenotype. Am J Med Genet. 1999;83(1):43-46. 10076883

[117] Seo GH, Kim TH, Chung JH. Antithyroid drugs and congenital malformations: a nationwide Korean cohort study. Ann Intern Med. 2018;168(6):405-413. 29357398 10.7326/M17-1398

[118] Andersen SL, Knøsgaard L, Olsen J, Vestergaard P, Andersen S. Maternal thyroid function, use of antithyroid drugs in early pregnancy, and birth defects. J Clin Endocrinol Metab. 2019;104(12):6040-6048. 31408173 10.1210/jc.2019-01343

[119] Andersen SL, Olsen J, Wu CS, Laurberg P. Severity of birth defects after propylthiouracil exposure in early pregnancy. Thyroid. 2014;24(10):1533-1540. 24963758 10.1089/thy.2014.0150 PMC4195247

[120] Andersen SL, Andersen S. Timing of shift in antithyroid drug therapy and birth defects. Thyroid. 2019;29(1):155-156. 30458113 10.1089/thy.2018.0621

[121] Azizi F, Amouzegar A, Tohidi M, et al. Increased remission rates after long-term methimazole therapy in patients with Graves’ disease: results of a randomized clinical trial. Thyroid. 2019;29(9):1192-1200. 31310160 10.1089/thy.2019.0180

[122] Reinwein D, Benker G, Lazarus JH, Alexander WD. A prospective randomized trial of antithyroid drug dose in Graves’ disease therapy. European Multicenter Study Group on Antithyroid Drug Treatment. J Clin Endocrinol Metab. 1993;76(6):1516-1521. 8501160 10.1210/jcem.76.6.8501160

[123] Sato S, Noh JY, Sato S, et al. Comparison of efficacy and adverse effects between methimazole 15 mg+inorganic iodine 38 mg/day and methimazole 30 mg/day as initial therapy for Graves’ disease patients with moderate to severe hyperthyroidism. Thyroid. 2015;25(1):43-50. 25178068 10.1089/thy.2014.0084

[124] Azizi F, Malboosbaf R. Safety of long-term antithyroid drug treatment? A systematic review. J Endocrinol Invest. 2019;42(11):1273-1283. 31134536 10.1007/s40618-019-01054-1

[125] Laurberg P, Berman DC, Andersen S, Bülow Pedersen I. Sustained control of Graves’ hyperthyroidism during long-term low-dose antithyroid drug therapy of patients with severe Graves’ orbitopathy. Thyroid. 2011;21(9):951-956. 21834677 10.1089/thy.2011.0039

[126] Azizi F, Ataie L, Hedayati M, Mehrabi Y, Sheikholeslami F. Effect of long-term continuous methimazole treatment of hyperthyroidism: comparison with radioiodine. Eur J Endocrinol. 2005;152(5):695-701. 15879354 10.1530/eje.1.01904

[127] Elbers L, Mourits M, Wiersinga W. Outcome of very long-term treatment with antithyroid drugs in Graves’ hyperthyroidism associated with Graves’ orbitopathy. Thyroid. 2011;21(3):279-283. 21190446 10.1089/thy.2010.0181

[128] Villagelin D, Romaldini JH, Santos RB, Milkos AB, Ward LS. Outcomes in relapsed Graves’ disease patients following radioiodine or prolonged low dose of methimazole treatment. Thyroid. 2015;25(12):1282-1290. 26414885 10.1089/thy.2015.0195

[129] Rivkees SA, Mattison DR. Ending propylthiouracil-induced liver failure in children. N Engl J Med. 2009;360(15):1574-1575. 10.1056/NEJMc0809750 19357418

[130] Biondi B, Bartalena L, Cooper DS, Hegedüs L, Laurberg P, Kahaly GJ. The 2015 European Thyroid Association Guidelines on diagnosis and treatment of endogenous subclinical hyperthyroidism. Eur Thyroid J. 2015;4(3):149-163. 26558232 10.1159/000438750 PMC4637513

[131] Gencer B, Collet TH, Virgini V, et al.; Thyroid Studies Collaboration. Subclinical thyroid dysfunction and the risk of heart failure events: an individual participant data analysis from 6 prospective cohorts. Circulation. 2012;126(9):1040-1049. 22821943 10.1161/CIRCULATIONAHA.112.096024 PMC3884576

[132] Collet TH, Gussekloo J, Bauer DC, et al.; Thyroid Studies Collaboration. Subclinical hyperthyroidism and the risk of coronary heart disease and mortality. Arch Intern Med. 2012;172(10):799-809. 22529182 10.1001/archinternmed.2012.402 PMC3872478

[133] Blum MR, Bauer DC, Collet TH, et al.; Thyroid Studies Collaboration. Subclinical thyroid dysfunction and fracture risk: a meta-analysis. JAMA. 2015;313(20):2055-2065. 26010634 10.1001/jama.2015.5161 PMC4729304

[134] Zhyzhneuskaya S, Addison C, Tsatlidis V, Weaver JU, Razvi S. The natural history of subclinical hyperthyroidism in Graves’ disease: the rule of thirds. Thyroid. 2016;26(6):765-769. 27090092 10.1089/thy.2015.0470

[135] Kahaly GJ, Dillmann WH. Thyroid hormone action in the heart. Endocr Rev. 2005;26(5):704-728. 15632316 10.1210/er.2003-0033

[136] Biondi B, Kahaly GJ. Cardiovascular involvement in patients with different causes of hyperthyroidism. Nat Rev Endocrinol. 2010;6(8):431-443. 20585347 10.1038/nrendo.2010.105

[137] Bartalena L, Baldeschi L, Dickinson A, et al.; European Group on Graves’ Orbitopathy (EUGOGO). Consensus statement of the European Group on Graves’ orbitopathy (EUGOGO) on management of GO. Eur J Endocrinol. 2008;158(3):273-285. 18299459 10.1530/EJE-07-0666

[138] McDermott MT, Kidd GS, Dodson LE Jr, Hofeldt FD. Radioiodine-induced thyroid storm. Case report and literature review. Am J Med. 1983;75(2):353-359. 6349350 10.1016/0002-9343(83)91217-2

[139] Burch HB, Solomon BL, Cooper DS, Ferguson P, Walpert N, Howard R. The effect of antithyroid drug pretreatment on acute changes in thyroid hormone levels after (131)I ablation for Graves’ disease. J Clin Endocrinol Metab. 2001;86(7):3016-3021. 11443161 10.1210/jcem.86.7.7639

[140] Ross DS . Radioiodine therapy for hyperthyroidism. N Engl J Med. 2011;364(6):542-550. 21306240 10.1056/NEJMct1007101

[141] Aung ET, Zammitt NN, Dover AR, Strachan MWJ, Seckl JR, Gibb FW. Predicting outcomes and complications following radioiodine therapy in Graves’ thyrotoxicosis. Clin Endocrinol (Oxf). 2019;90(1):192-199. 30291728 10.1111/cen.13873

[142] Andrade VA, Gross JL, Maia AL. Effect of methimazole pretreatment on serum thyroid hormone levels after radioactive treatment in Graves’ hyperthyroidism. J Clin Endocrinol Metab. 1999;84(11):4012-4016. 10566642 10.1210/jcem.84.11.6149

[143] Teng CJ, Hu YW, Chen SC, et al. Use of radioactive iodine for thyroid cancer and risk of second primary malignancy: a nationwide population-based study. J Natl Cancer Inst. 2016;108(2):1-8. 10.1093/jnci/djv314 26538627

[144] Metso S, Auvinen A, Huhtala H, Salmi J, Oksala H, Jaatinen P. Increased cancer incidence after radioiodine treatment for hyperthyroidism. Cancer. 2007;109(10):1972-1979. 17393376 10.1002/cncr.22635

[145] Hieu TT, Russell AW, Cuneo R, et al. Cancer risk after medical exposure to radioactive iodine in benign thyroid diseases: a meta-analysis. Endocr Relat Cancer. 2012;19(5):645-655. 22851687 10.1530/ERC-12-0176

[146] Ryödi E, Metso S, Jaatinen P, et al. Cancer incidence and mortality in patients treated either with RAI or thyroidectomy for hyperthyroidism. J Clin Endocrinol Metab. 2015;100(10):3710-3717. 26262435 10.1210/jc.2015-1874

[147] Kitahara CM, Berrington de Gonzalez A, Bouville A, et al. Association of radioactive iodine treatment with cancer mortality in patients with hyperthyroidism. JAMA Intern Med. 2019;179(8):1034-1042. 31260066 10.1001/jamainternmed.2019.0981 PMC6604114

[148] Kitahara CM, Preston DL, Sosa JA, Berrington de Gonzalez A. Association of radioactive iodine, antithyroid drug, and surgical treatments with solid cancer mortality in patients with hyperthyroidism. JAMA Netw Open. 2020;3(7):e209660. 32701159 10.1001/jamanetworkopen.2020.9660 PMC7378755

[149] Gronich N, Lavi I, Rennert G, Saliba W. Cancer risk after radioactive iodine treatment for hyperthyroidism: a cohort study. Thyroid. 2020;30(2):243-250. 31880205 10.1089/thy.2019.0205

[150] De Leo S, Lee SY, Braverman LE. Hyperthyroidism. Lancet. 2016;388(10047):906-918. 27038492 10.1016/S0140-6736(16)00278-6 PMC5014602

[151] Randle RW, Bates MF, Long KL, Pitt SC, Schneider DF, Sippel RS. Impact of potassium iodide on thyroidectomy for Graves’ disease: implications for safety and operative difficulty. Surgery. 2018;163(1):68-72. 29108701 10.1016/j.surg.2017.03.030 PMC5736456

[152] Genovese BM, Noureldine SI, Gleeson EM, Tufano RP, Kandil E. What is the best definitive treatment for Graves’ disease? A systematic review of the existing literature. Ann Surg Oncol. 2013;20(2):660-667. 22956065 10.1245/s10434-012-2606-x

[153] Kandil E, Noureldine SI, Abbas A, et al. The impact of surgical volume on patient outcomes following thyroid surgery. Surgery. 2013;154(6):1346-1352; discussion 52-53. 24238052 10.1016/j.surg.2013.04.068

[154] Hauch A, Al-Qurayshi Z, Randolph G, Kandil E. Total thyroidectomy is associated with increased risk of complications for low- and high-volume surgeons. Ann Surg Oncol. 2014;21(12):3844-3852. 24943236 10.1245/s10434-014-3846-8

[155] Dralle H . Surgical assessment of complications after thyroid gland operations. Chirurg. 2015;86(1):70-77. 25234502 10.1007/s00104-014-2819-6

[156] Oltmann SC, Brekke AV, Schneider DF, Schaefer SC, Chen H, Sippel RS. Preventing postoperative hypocalcemia in patients with Graves disease: a prospective study. Ann Surg Oncol. 2015;22(3):952-958. 25212835 10.1245/s10434-014-4077-8 PMC4318765

[157] Xing T, Hu Y, Wang B, Zhu J. Role of oral calcium supplementation alone or with vitamin D in preventing post-thyroidectomy hypocalcaemia: a meta-analysis. Medicine (Baltimore). 2019;98(8):e14455. 30813146 10.1097/MD.0000000000014455 PMC6407934

[158] Di Donna V, Santoro MG, de Waure C, et al. A new strategy to estimate levothyroxine requirement after total thyroidectomy for benign thyroid disease. Thyroid. 2014;24(12):1759-1764. 25268754 10.1089/thy.2014.0111

[159] Elfenbein DM, Schaefer S, Shumway C, Chen H, Sippel RS, Schneider DF. Prospective intervention of a novel levothyroxine dosing protocol based on body mass index after thyroidectomy. J Am Coll Surg. 2016;222(1):83-88. 26584573 10.1016/j.jamcollsurg.2015.10.005 PMC4698011

[160] Taylor PN, Zhang L, Lee RWJ, et al. New insights into the pathogenesis and nonsurgical management of Graves orbitopathy. Nat Rev Endocrinol. 2020;16(2):104-116. 31889140 10.1038/s41574-019-0305-4

[161] Diana T, Ponto KA, Kahaly GJ. Thyrotropin receptor antibodies and Graves’ orbitopathy. [Published online on August 04, 2020]. J Endocrinol Invest. 2020. Doi: 10.1007/s40618-020-01380-9 PMC8310479 32749654

[162] Wiersinga WM, Perros P, Kahaly GJ, et al. Clinical assessment of patients with Graves’ orbitopathy: the European Group on Graves’ Orbitopathy recommendations to generalists, specialists and clinical researchers. Eur J Endocrinol. 2006;155(3):387-389. 16914591 10.1530/eje.1.02230

[163] Kahaly GJ, Diana T, Kanitz M, et al. Prospective trial of functional thyrotropin receptor antibodies in Graves disease. J Clin Endocrinol Metab. 2020;105(4):e1006-14. 10.1210/clinem/dgz292 PMC7067543 31865369

[164] Kahaly GJ, Wüster C, Olivo PD, Diana T. High titers of thyrotropin receptor antibodies are associated with orbitopathy in patients with Graves disease. J Clin Endocrinol Metab. 2019;104(7):2561-2568. 30753531 10.1210/jc.2018-02705

[165] Marcocci C, Kahaly GJ, Krassas GE, et al.; European Group on Graves’ Orbitopathy. Selenium and the course of mild Graves’ orbitopathy. N Engl J Med. 2011;364(20):1920-1931. 21591944 10.1056/NEJMoa1012985

[166] Kahaly GJ . Management of moderately severe graves’ orbitopathy. In: Graves’ Orbitopathy - A Multidisciplinary Approach Questions and Answers. 3rd ed. Basel: Karger; 2017.

[167] Jespersen S, Nygaard B, Kristensen LØ. Methylprednisolone pulse treatment of Graves’ ophthalmopathy is not associated with secondary adrenocortical insufficiency. Eur Thyroid J. 2015;4(4):222-225. 26835424 10.1159/000440834 PMC4716422

[168] Zang S, Ponto KA, Kahaly GJ. Clinical review: intravenous glucocorticoids for Graves’ orbitopathy: efficacy and morbidity. J Clin Endocrinol Metab. 2011;96(2):320-332. 21239515 10.1210/jc.2010-1962

[169] Zang S, Ponto KA, Pitz S, Kahaly GJ. Dose of intravenous steroids and therapy outcome in Graves’ orbitopathy. J Endocrinol Invest. 2011;34(11):876-880. 22322535 10.1007/BF03346732

[170] Tanda ML, Bartalena L. Efficacy and safety of orbital radiotherapy for graves’ orbitopathy. J Clin Endocrinol Metab. 2012;97(11):3857-3865. 22962421 10.1210/jc.2012-2758

[171] Wiersinga WM, Kahaly GJ Graves’ Orbitopathy a Multidisciplinary Approach. 3rd ed. Basel: Karger; 2017.

[172] Bahn RS . Graves’ ophthalmopathy. N Engl J Med. 2010;362(8):726-738. 20181974 10.1056/NEJMra0905750 PMC3902010

[173] Moshkelgosha S, So PW, Deasy N, Diaz-Cano S, Banga JP. Cutting edge: retrobulbar inflammation, adipogenesis, and acute orbital congestion in a preclinical female mouse model of Graves’ orbitopathy induced by thyrotropin receptor plasmid-in vivo electroporation. Endocrinology. 2013;154(9):3008-3015. 23900776 10.1210/en.2013-1576

[174] Faßbender J, Holthoff HP, Li Z, Ungerer M. Therapeutic effects of short cyclic and combined epitope peptides in a long-term model of Graves’ disease and orbitopathy. Thyroid. 2019;29(2):258-267. 30618332 10.1089/thy.2018.0326

[175] Holthoff HP, Goebel S, Li Z, et al. Prolonged TSH receptor A subunit immunization of female mice leads to a long-term model of Graves’ disease, tachycardia, and cardiac hypertrophy. Endocrinology. 2015;156(4):1577-1589. 25562617 10.1210/en.2014-1813

[176] Holthoff HP, Li Z, Faßbender J, et al. Cyclic peptides for effective treatment in a long-term model of Graves disease and orbitopathy in female mice. Endocrinology. 2017;158(7):2376-2390. 28368444 10.1210/en.2016-1845

[177] Hai YP, Lee ACH, Frommer L, Diana T, Kahaly GJ. Immunohistochemical analysis of human orbital tissue in Graves’ orbitopathy. J Endocrinol Invest. 2020;43(2):123-137. 31538314 10.1007/s40618-019-01116-4

[178] Zhang L, Baker G, Janus D, Paddon CA, Fuhrer D, Ludgate M. Biological effects of thyrotropin receptor activation on human orbital preadipocytes. Invest Ophthalmol Vis Sci. 2006;47(12):5197-5203. 17122103 10.1167/iovs.06-0596 PMC1892592

[179] Wang Y, Smith TJ. Current concepts in the molecular pathogenesis of thyroid-associated ophthalmopathy. Invest Ophthalmol Vis Sci. 2014;55(3):1735-1748. 24651704 10.1167/iovs.14-14002 PMC3968932

[180] Weightman DR, Perros P, Sherif IH, Kendall-Taylor P. Autoantibodies to IGF-1 binding sites in thyroid associated ophthalmopathy. Autoimmunity. 1993;16(4):251-257. 7517705 10.3109/08916939309014643

[181] Tsui S, Naik V, Hoa N, et al. Evidence for an association between thyroid-stimulating hormone and insulin-like growth factor 1 receptors: a tale of two antigens implicated in Graves’ disease. J Immunol. 2008;181(6):4397-4405. 18768899 10.4049/jimmunol.181.6.4397 PMC2775538

[182] Douglas RS, Gianoukakis AG, Kamat S, Smith TJ. Aberrant expression of the insulin-like growth factor-1 receptor by T cells from patients with Graves’ disease may carry functional consequences for disease pathogenesis. J Immunol. 2007;178(5):3281-3287. 17312178 10.4049/jimmunol.178.5.3281

[183] Smith TJ, Janssen J. Insulin-like growth factor-I receptor and thyroid-associated ophthalmopathy. Endocr Rev. 2019;40(1):236-267. 30215690 10.1210/er.2018-00066 PMC6338478

[184] Minich WB, Dehina N, Welsink T, et al. Autoantibodies to the IGF1 receptor in Graves’ orbitopathy. J Clin Endocrinol Metab. 2013;98(2):752-760. 23264397 10.1210/jc.2012-1771

[185] Marinò M, Rotondo Dottore G, Ionni I, et al. Serum antibodies against the insulin-like growth factor-1 receptor (IGF-1R) in Graves’ disease and Graves’ orbitopathy. J Endocrinol Invest. 2019;42(4):471-480. 30132285 10.1007/s40618-018-0943-8

[186] Krieger CC, Place RF, Bevilacqua C, et al. TSH/IGF-1 receptor cross talk in Graves’ ophthalmopathy pathogenesis. J Clin Endocrinol Metab. 2016;101(6):2340-2347. 27043163 10.1210/jc.2016-1315 PMC4891793

[187] Krieger CC, Perry JD, Morgan SJ, Kahaly GJ, Gershengorn MC. TSH/IGF-1 receptor cross-talk rapidly activates extracellular signal-regulated kinases in multiple cell types. Endocrinology. 2017;158(10):3676-3683. 28938449 10.1210/en.2017-00528 PMC5659693

[188] Krieger CC, Boutin A, Jang D, et al. Arrestin-β-1 physically scaffolds TSH and IGF1 receptors to enable crosstalk. Endocrinology. 2019;160(6):1468-1479. 31127272 10.1210/en.2019-00055 PMC6542485

[189] Marcus-Samuels B, Krieger CC, Boutin A, Kahaly GJ, Neumann S, Gershengorn MC. Evidence that Graves’ ophthalmopathy immunoglobulins do not directly activate IGF-1 receptors. Thyroid. 2018;28(5):650-655. 29631510 10.1089/thy.2018.0089 PMC5952334

[190] Pritchard J, Han R, Horst N, Cruikshank WW, Smith TJ. Immunoglobulin activation of T cell chemoattractant expression in fibroblasts from patients with Graves’ disease is mediated through the insulin-like growth factor I receptor pathway. J Immunol. 2003;170(12):6348-6354. 12794168 10.4049/jimmunol.170.12.6348

[191] Chen H, Shan SJ, Mester T, Wei YH, Douglas RS. TSH-mediated TNFα production in human fibrocytes is inhibited by teprotumumab, an IGF-1R antagonist. PLoS One. 2015;10(6):e0130322. 26087256 10.1371/journal.pone.0130322 PMC4472723

[192] Chen H, Mester T, Raychaudhuri N, et al. Teprotumumab, an IGF-1R blocking monoclonal antibody inhibits TSH and IGF-1 action in fibrocytes. J Clin Endocrinol Metab. 2014;99(9):E1635-E1640. 24878056 10.1210/jc.2014-1580 PMC4154099

[193] Smith TJ, Kahaly GJ, Ezra DG, et al. Teprotumumab for thyroid-associated ophthalmopathy. N Engl J Med. 2017;376(18):1748-1761. 28467880 10.1056/NEJMoa1614949 PMC5718164

[194] Douglas RS, Kahaly GJ, Patel A, et al. Teprotumumab for the treatment of active thyroid eye disease. N Engl J Med. 2020;382(4):341-352. 31971679 10.1056/NEJMoa1910434

[195] Smith TJ, Bartalena L. Will biological agents supplant systemic glucocorticoids as the first-line treatment for thyroid-associated ophthalmopathy? Eur J Endocrinol. 2019;181(5):D27-D43. 31370005 10.1530/EJE-19-0389 PMC7398270

[196] Douglas RS, Kahaly GJ. Teprotumumab for active thyroid eye disease. Reply. N Engl J Med. 2020;382(20):1959-1960. 10.1056/NEJMc2002754 32402172

[197] Bartalena L, Fatourechi V. Extrathyroidal manifestations of Graves’ disease: a 2014 update. J Endocrinol Invest. 2014;37(8):691-700. 24913238 10.1007/s40618-014-0097-2

[198] Fatourechi V . Thyroid dermopathy and acropachy. Best Pract Res Clin Endocrinol Metab. 2012;26(4):553-565. 22863396 10.1016/j.beem.2011.10.001

[199] Fatourechi V, Ahmed DD, Schwartz KM. Thyroid acropachy: report of 40 patients treated at a single institution in a 26-year period. J Clin Endocrinol Metab. 2002;87(12):5435-5441. 12466333 10.1210/jc.2002-020746

[200] Schwartz KM, Fatourechi V, Ahmed DD, Pond GR. Dermopathy of Graves’ disease (pretibial myxedema): long-term outcome. J Clin Endocrinol Metab. 2002;87(2):438-446. 11836263 10.1210/jcem.87.2.8220

[201] Okosieme OE, Taylor PN, Evans C, et al. Primary therapy of Graves’ disease and cardiovascular morbidity and mortality: a linked-record cohort study. Lancet Diabetes Endocrinol. 2019;7(4):278-287. 30827829 10.1016/S2213-8587(19)30059-2

[202] Akamizu T . Thyroid storm: a Japanese perspective. Thyroid. 2018;28(1):32-40. 28899229 10.1089/thy.2017.0243 PMC5770119

[203] Bartalena L, Chiovato L, Marcocci C, Vitti P, Piantanida E, Tanda ML. Management of Graves’ hyperthyroidism and orbitopathy in time of COVID-19 pandemic. J Endocrinol Invest. 2020;43(8):1149-1151. 32441005 10.1007/s40618-020-01293-7 PMC7241069

[204] Kaiser UB, Mirmira RG, Stewart PM. Our response to COVID-19 as Endocrinologists and Diabetologists. J Clin Endocrinol Metab. 2020;105(5):1299-1301. 10.1210/clinem/dgaa148 PMC7108679 32232480

[205] Puig-Domingo M, Marazuela M, Giustina A. COVID-19 and endocrine diseases. A statement from the European Society of Endocrinology. Endocrine. 2020;68(1):2-5. 32279224 10.1007/s12020-020-02294-5 PMC7150529

[206] Kahaly GJ, Pitz S, Hommel G, Dittmar M. Randomized, single blind trial of intravenous versus oral steroid monotherapy in Graves’ orbitopathy. J Clin Endocrinol Metab. 2005;90(9):5234-5240. 15998777 10.1210/jc.2005-0148

[207] Paridaens D, van den Bosch WA, van der Loos TL, Krenning EP, van Hagen PM. The effect of etanercept on Graves’ ophthalmopathy: a pilot study. Eye (Lond). 2005;19(12):1286-1289. 15550932 10.1038/sj.eye.6701768

[208] Ayabe R, Rootman DB, Hwang CJ, Ben-Artzi A, Goldberg R. Adalimumab as steroid-sparing treatment of inflammatory-stage thyroid eye disease. Ophthalmic Plast Reconstr Surg. 2014;30(5):415-419. 24978425 10.1097/IOP.0000000000000211

[209] Allison AC . Mechanisms of action of mycophenolate mofetil in preventing chronic rejection. Transplant Proc. 2002;34(7):2863-2866. 12431636 10.1016/s0041-1345(02)03538-8

[210] Kahaly GJ, Riedl M, König J, et al.; European Group on Graves’ Orbitopathy (EUGOGO). Mycophenolate plus methylprednisolone versus methylprednisolone alone in active, moderate-to-severe Graves’ orbitopathy (MINGO): a randomised, observer-masked, multicentre trial. Lancet Diabetes Endocrinol. 2018;6(4):287-298. 29396246 10.1016/S2213-8587(18)30020-2

[211] Ye X, Bo X, Hu X, et al. Efficacy and safety of mycophenolate mofetil in patients with active moderate-to-severe Graves’ orbitopathy. Clin Endocrinol (Oxf). 2017;86(2):247-255. 27484048 10.1111/cen.13170

[212] Lee ACH, Riedl M, Frommer L, Diana T, Kahaly GJ. Systemic safety analysis of mycophenolate in Graves’ orbitopathy. J Endocrinol Invest. 2020;43(6):767-777. 31834613 10.1007/s40618-019-01161-z

[213] Riedl M, Kuhn A, Krämer I, Kolbe E, Kahaly GJ. Prospective, systematically recorded mycophenolate safety data in Graves’ orbitopathy. J Endocrinol Invest. 2016;39(6):687-694. 26886940 10.1007/s40618-016-0441-9

[214] Sanders P, Young S, Sanders J, et al. Crystal structure of the TSH receptor (TSHR) bound to a blocking-type TSHR autoantibody. J Mol Endocrinol. 2011;46(2):81-99. 21247981 10.1530/JME-10-0127

[215] Furmaniak J, Sanders J, Rees Smith B. Blocking type TSH receptor antibodies. Auto Immun Highlights. 2013;4(1):11-26. 26000138 10.1007/s13317-012-0028-1 PMC4389084

[216] Marcinkowski P, Hoyer I, Specker E, et al. A new highly thyrotropin receptor-selective small-molecule antagonist with potential for the treatment of Graves’ orbitopathy. Thyroid. 2019;29(1):111-123. 30351237 10.1089/thy.2018.0349

[217] Jansson L, Vrolix K, Jahraus A, Martin KF, Wraith DC. Immunotherapy with apitopes blocks the immune response to TSH receptor in HLA-DR transgenic mice. Endocrinology. 2018;159(9):3446-3457. 30099489 10.1210/en.2018-00306

[218] Pearce SHS, Dayan C, Wraith DC, et al. Antigen-specific immunotherapy with thyrotropin receptor peptides in Graves’ hyperthyroidism: a phase I study. Thyroid. 2019;29(7):1003-1011. 31194638 10.1089/thy.2019.0036 PMC6648194

[219] Cordoba F, Wieczorek G, Audet M, et al. A novel, blocking, Fc-silent anti-CD40 monoclonal antibody prolongs nonhuman primate renal allograft survival in the absence of B cell depletion. Am J Transplant. 2015;15(11):2825-2836. 26139432 10.1111/ajt.13377

[220] Ristov J, Espie P, Ulrich P, et al. Characterization of the in vitro and in vivo properties of CFZ533, a blocking and non-depleting anti-CD40 monoclonal antibody. Am J Transplant. 2018;18(12):2895-2904. 29665205 10.1111/ajt.14872

[221] Kahaly GJ, Stan MN, Frommer L, et al. A novel anti-CD40 monoclonal antibody, iscalimab, for control of graves hyperthyroidism-a proof-of-concept trial. J Clin Endocrinol Metab. 2020;105(3):696-704. 10.1210/clinem/dgz013 31512728

[222] El Fassi D, Nielsen CH, Bonnema SJ, Hasselbalch HC, Hegedüs L. B lymphocyte depletion with the monoclonal antibody rituximab in Graves’ disease: a controlled pilot study. J Clin Endocrinol Metab. 2007;92(5):1769-1772. 17284622 10.1210/jc.2006-2388

[223] Stan MN, Garrity JA, Carranza Leon BG, Prabin T, Bradley EA, Bahn RS. Randomized controlled trial of rituximab in patients with Graves’ orbitopathy. J Clin Endocrinol Metab. 2015;100(2):432-441. 25343233 10.1210/jc.2014-2572 PMC4318907

[224] Salvi M, Vannucchi G, Currò N, et al. Efficacy of B-cell targeted therapy with rituximab in patients with active moderate to severe Graves’ orbitopathy: a randomized controlled study. J Clin Endocrinol Metab. 2015;100(2):422-431. 25494967 10.1210/jc.2014-3014 PMC4318899

[225] Stan MN, Salvi M. MANAGEMENT OF ENDOCRINE DISEASE: rituximab therapy for Graves’ orbitopathy - lessons from randomized control trials. Eur J Endocrinol. 2017;176(2):R101-R109. 27760790 10.1530/EJE-16-0552

[226] Pérez-Moreiras JV, Alvarez-López A, Gómez EC. Treatment of active corticosteroid-resistant graves’ orbitopathy. Ophthalmic Plast Reconstr Surg. 2014;30(2):162-167. 24503568 10.1097/IOP.0000000000000037

[227] Perez-Moreiras JV, Gomez-Reino JJ, Maneiro JR, et al.; Tocilizumab in Graves Orbitopathy Study Group. Efficacy of tocilizumab in patients with moderate-to-severe corticosteroid-resistant Graves orbitopathy: a randomized clinical trial. Am J Ophthalmol. 2018;195(1):181-190. 30081019 10.1016/j.ajo.2018.07.038

